# Hyperthermia exaggerates exercise-induced aggregation of blood platelets

**DOI:** 10.1186/2046-7648-4-S1-A153

**Published:** 2015-09-14

**Authors:** Jung-Hyun Kim, Tianzhou Wu, Raymond Roberge, Aitor Coca

**Affiliations:** 1 National Personal Protective Technology Laboratory, National Institute for Occupational Safety and Health, Centers for Disease Control and Prevention, Pittsburgh, PA, USA

## Introduction

Acute exposure to exertional exercise/heavy physical work often triggers cardiovascular events in which exercise-induced platelet aggregation, blood coagulation, and disruption in fibrinolysis may adversely affect atherothrombotic disease. Elevated body temperature, commonly accompanied with prolonged exercise, was suggested as an auxiliary factor for exercise-induced platelet aggregation [1]. Recent studies also showed platelet hyperaggregation following firefighting activities combining heavy physical work and heat stress [2], [3]. However, the influence of hyperthermia separated from physical exercise impact on platelet aggregation is unclear.

## Methods

Twelve healthy men; age 22.8 (1.3) years and VO_2max _56.8 (6.2) ml.kg^-1^.min^-1^, underwent three experimental trials: exercise hyperthermia (ExHT), passive hyperthermia (PaHT), and control exercise (CONT). Subjects performed a treadmill exercise at 60 % VO_2max _in the heat (35 °C, 50 % RH) until their rectal temperature (T_re_) increased 1.5 °C above the resting baseline (ExHT) or performed a control exercise at the same intensity and duration according to ExHT in a cooler condition (23 °C, 50 % RH) (CONT). In PaHT, subjects were passively heated using a water garment (45 °C) in the heat (45 °C, 50 % RH) until T_re _increased 1.5 °C above baseline. Platelet aggregation was assessed from antecubital venous blood collected during baseline (Base), end-trial (End), and again following 1 hour of passive recovery (Rec) (23 °C, 50 % RH), using a platelet function analyser providing a closure time (CT: second) through an in-vitro simulation of platelet adhesion, activation, and aggregation. Decreased CT is an indicative of increased platelet aggregation. Dependent variables were analysed using a two-way repeated measures ANOVA.

## Results

Under the study conditions, T_re _(*F *= 13.2, *p*<0.001) and skin temperature (*F *= 97.3, *p*<0.001) increased significantly in ExHT and PaHT compared to CONT, whereas heart rate was significantly higher in ExHT and CONT compared to PaHT (*F *= 40.0, *p*<0.001). CT in exposure to Collagen/ADP showed a decreasing trend over time in ExHT and PaHT and significantly differed from CONT at Rec (*F *= 7.6, *p *= 0.008). CT in exposure to Collagen/Epinephrine showed a similar response to Collagen/ADP, but did not significantly differ among conditions (*F *= 3.5, *p *= 0.075), though CT in ExHT significantly decreased at End compared to CT in CONT (*p *= 0.046).

## Discussion

Moderate exercise in the heat (ExHT) significantly elevated platelet aggregation as indicated by decreased CT whereas CT was not altered in non-hyperthermia exercise condition (CONT). PaHT showed an overall decreasing trend of CT toward End and Rec, but its impact on platelet aggregation was not significant in response to C/EPI in this study.

## Conclusion

It was concluded that hyperthermia exaggerates exercise-induced platelet aggregation as an auxiliary factor, but the effect of hyperthermia alone on platelet aggregation in young, healthy subjects is minimal. Further research is warranted to investigate a physiological mechanism responsible for hyperthermia induced-platelet hyperreactivity.

## Disclaimer

The findings and conclusions of this abstract are those of the authors and do not necessarily reflect the views of the National Institute for Occupational Safety and Health.
